# List of Diseases from the 20th of April to the 20th of May; Being the Result of the Public and Private Practice of a Physician at the West End of the Town

**Published:** 1799-06

**Authors:** 


*List of Diseases from the 20th of April to the 20th of May; being the Result of the Public and Private Practice of a Physician at the West End of the Town.*

FEBRILE DISEASES.

| | No. of Cases. | | No. of Cases. |
|---|---|---|---|
| Catarrh | 13 | Slow Fever, and Hectic | 9 |
| Acute Rheumatism | 5 | Child-bed-fever | 3 |
| Hooping-Cough | 6 | Febrile Diseases of Infants | 9 |
| Measles | 5 | Aphthous Sore-throat | 3 |
| Scarlatina | 4 | Hæmoptoe | 1 |
| Chicken-pox | 1 | Hæmatemesis | 1 |
| Contagious malignant Fever | 3 | Quotidian | 1 |
| | | Tertian | 1 |

CHRONIC

| CHRONIC DISEASES. | No. of Cases. | | No. of Cases. |
|---|---|---|---|
| Cough and Dyſpnœa | 36 | Chloroſis, &c. | 7 |
| Pulmonary Conſumption | 7 | Menorrhagia | 5 |
| Pleurodyne | 4 | Abortion | 2 |
| Chronic Rheumatiſm | 15 | Fluor albus | 2 |
| Aſthenia | 21 | Dyſury and Gravel | 3 |
| Dropſy | 7 | Hæmorrhoids | 2 |
| Scrophula | 5 | Tabes Meſenterica | 6 |
| Rachitis | 2 | Scirrhus | 4 |
| Cephalæa | 8 | Tooth raſh, &c. | 6 |
| Epilepſy | 1 | Itch and Prurigo | 15 |
| Vertigo | 2 | Lepra, and Scaly Tetter | 5 |
| Dyſpepſia | 10 | Intertrigo | 2 |
| Pains of the Stomach and Bowels | 14 | Acne | 5 |
| Diarrhœa | 6 | Nettle-raſh | 1 |
| Conſtipation | 2 | Purpura | 2 |
| | | Erythema | 1 |
| | | Porrigo | 8 |

